# Bibliometric Mapping of 40 Years of AI in Nursing: Trends, Collaborations, and Research Hotspots Worldwide

**DOI:** 10.1155/jonm/6154880

**Published:** 2026-01-31

**Authors:** Mualla Dikmen, Filiz Elmalı

**Affiliations:** ^1^ Independent Researcher, Elazığ, Türkiye; ^2^ Department of Elementary Education, Faculty of Education, Fırat University, Elazığ, Türkiye, firat.edu.tr

**Keywords:** artificial intelligence, bibliometric, nursing

## Abstract

**Background:**

Artificial intelligence (AI) has increasingly influenced healthcare, yet its adoption in nursing research remains underexplored. Although interest in AI applications, such as robotics, decision support, and machine learning, is growing, a comprehensive bibliometric mapping of global scholarship in nursing remains lacking.

**Objective:**

To examine the global trends, intellectual structures, and thematic evolution of AI‐related research in nursing from 1984 to 2025 (through May 2025) and to identify key authors, institutions, and research foci.

**Methods:**

A systematic search was conducted in the Web of Science Core Collection using predefined title‐based keywords (“nurse” OR “nursing”) AND (“artificial intelligence” OR “machine learning” OR “deep learning” OR “robotic∗” OR “chatbot∗” OR “neural network∗”). Studies were included if they explicitly addressed nursing practice, management, education, or research applications of AI. Two independent reviewers screened all records by title and abstract to confirm nursing relevance using predefined inclusion and exclusion criteria. This yielded 799 records; after removing duplicates and uncited papers, 530 publications were retained as nursing‐related. Bibliometric analysis employed performance and science‐mapping techniques via VOSviewer and Biblioshiny. Indicators included publication trends, citation distributions, keyword co‐occurrence, authorship patterns, and collaborations.

**Results:**

The number of publications increased substantially after 2018, with the United States, China, and the United Kingdom being the most productive countries. Dominant research themes included robotics in elder care, clinical decision support systems, and nursing education enhanced by AI tools. Coauthorship analysis revealed limited international collaboration, and keyword mapping identified “robotics,” “machine learning,” and “nursing education” as leading focal points.

**Conclusions:**

Despite decades of development, AI remains an emerging and underutilized area within nursing research. The rapid growth of publications in recent years signals expanding interest, yet the field lacks consolidated efforts and cohesive global collaboration. Greater interdisciplinary and international engagement is needed to accelerate innovation.

## 1. Introduction

Artificial intelligence (AI) has gained remarkable visibility across healthcare research, driven by exponential growth in data generation and improved computational capacity. Although many domain‐specific reviews outline how machine learning [1], natural language processing [2], and related techniques assist diagnosis, treatment [3], and clinical workflows [4], these accounts do not capture the broader evolution of AI scholarship within nursing. As AI‐related publications continue to expand, understanding how the nursing discipline contributes to, collaborates within, and navigates this emerging field has become increasingly important. This requires methodological tools that move beyond narrative reviews toward reproducible, data‐driven bibliometric approaches.

Despite the overall growth of AI in healthcare, the incorporation of AI within nursing scholarship remains comparatively modest. While tens of thousands of AI‐focused studies exist across medicine and health informatics, only a few hundred explicitly address AI within nursing in major scholarly databases such as the Web of Science (WoS) [5]. This discrepancy signals a need to map how nursing engages with AI, not in terms of clinical mechanisms but in terms of research output, citation patterns, thematic clusters, and collaboration dynamics. Such a mapping can only be achieved through a systematic bibliometric framework.

### 1.1. Background and Research Gap

Existing research on AI in nursing primarily focuses on specific subdomains such as robotic caregiving [6], educational technologies [7], or decision support tools [8]. These studies shed light on focused application areas but do not systematically examine how nursing‐related AI research evolves across decades, which topics gain prominence, or how authors and institutions collaborate over time. Furthermore, narrative reviews and scoping studies [9, 10] tend to emphasize technological capabilities while offering limited insights into structural publication trends or intellectual linkages within the field.

Recent bibliometric contributions represent valuable initial steps. For example, Shi et al. [11] mapped global AI‐related nursing outputs and documented leading countries, journals, and topic clusters, while Çiçek‐Korkmaz [5] identified a sharp increase in publication and citation activity after 2018. However, these studies have methodological constraints, including narrow keyword retrieval, limited citation‐based refinement, insufficient exploration of coauthorship and institutional networks, and relatively short temporal coverage. Many do not treat nursing as a distinct conceptual and professional category, instead clustering it within broader health informatics terminology. Consequently, the current evidence base remains fragmented and does not provide a comprehensive understanding of AI in nursing from a long‐term perspective.

Taken together, the literature demonstrates a need for methodologically transparent and longitudinal bibliometric mapping that clearly articulates retrieval criteria, database parameters, screening rules, and analytical procedures. Bibliometric methods offer an analytical advantage for understanding the development of interdisciplinary and rapidly expanding fields such as AI in nursing. By quantifying publication volumes, citation trajectories, coauthorship patterns, and keyword co‐occurrence networks, bibliometric analysis provides structured insights into the evolution of a research domain [12]. Unlike narrative reviews, which may privilege selected studies or applications, bibliometrics enables a comprehensive and reproducible examination of the knowledge base [13].

These methodological strengths directly address the limitations highlighted by narrative assessments of AI applications in nursing. Rather than describing what AI can do in practice, bibliometrics provides systematic evidence of how nursing‐related AI research has evolved and where new contributions are likely to emerge.

### 1.2. Purpose and Contribution of the Present Study

The purpose of this study is to conduct a longitudinal, four‐decade bibliometric mapping of AI‐related nursing research indexed in the WoS Core Collection to date. The study employs a dual keyword strategy that integrates both nursing‐specific and AI‐specific concepts, enabling a balanced retrieval of publications positioned at the intersection of the two fields. All retrieval decisions, screening criteria, and database parameters are applied transparently to ensure methodological reproducibility. Acknowledging the existence of comparable long‐term bibliometric analyses, systematic review, and meta‐analysis tracking 40 years of progress in AI within broader health systems [14, 15], this work stands as one of the most comprehensive investigations focused specifically on the nursing discipline’s output and internal intellectual structure using WoS data.

The WoS Core Collection is selected for its high‐quality citation metadata, structured indexing approach, and established suitability for network‐based bibliometric analysis [16, 17]. Although historical records show that early AI‐related nursing study began around 1979, the first article to accumulate citations appeared in 1984 [5, 11]. This empirical marker provides a logical and evidence‐based starting point for longitudinal bibliometric analysis spanning 1984–2025 (through May 2025). Indeed, mapping techniques aim to ensure that the distance between items reflects the strength of the relationship [12]. Therefore, studies with zero citations (zero relationship strength) are not included in the mapping because they do not contribute significantly to the network structure.

This study advances the field in several important ways. It expands beyond earlier bibliometric research by offering broader temporal coverage, more robust retrieval strategies, and integrated citation and network analyses. It provides novel insights into the specific evolution of AI research within nursing, differentiating it from the broader health informatics field, which is critical given the methodological heterogeneity observed across diverse clinical applications of AI [15, 18]. It identifies the most influential contributors, institutions, and countries and maps the thematic evolution of AI within the nursing discipline across 4 decades.

## 2. Methods

This study is a bibliometric analysis of the scientific literature on AI in nursing, conducted using systematic identification, screening, eligibility, and inclusion procedures in accordance with PRISMA 2020 reporting guidelines. The study provides a comprehensive overview of the existing literature on the use of AI in nursing within the WoS Core Collection. Without applying any restrictions on publication year, the study analyzed research related to the use of AI in nursing, based on the scientific data provided by WoS. The analysis of international studies indexed in WoS was carried out using bibliometric mapping techniques. Data retrieved from the literature were analyzed to identify the most frequently used keywords, the most cited publications, journals, countries, and authors. These components were then visualized using relational mapping within the framework of bibliometric analysis. Our methodological framework aligns with the recent bibliometric and science mapping studies in related interdisciplinary fields [19]. These studies emphasized rigorous data retrieval from the WoS Core Collection, standardized keyword selection, and science mapping using Visual Similarity Viewer (VOSviewer) to visualize research hotspots and collaborations.

### 2.1. Dataset Construction

The dataset for the current study was obtained through a systematic search conducted in the WoS database on May 27, 2025. The study utilized seven citation indexes available in the WoS:

• Conference Proceedings Citation Index–Social Science & Humanities (CPCI‐SSH),
• Conference Proceedings Citation Index–Science (CPCI‐S),
• Social Sciences Citation Index (SSCI),
• Emerging Sources Citation Index (ESCI),
• Science Citation Index Expanded (SCI‐Expanded),
• Arts & Humanities Citation Index (A&HCI), and
• Book Citation Index–Social Sciences & Humanities (BKCI‐SSH).

**FIGURE 1:**
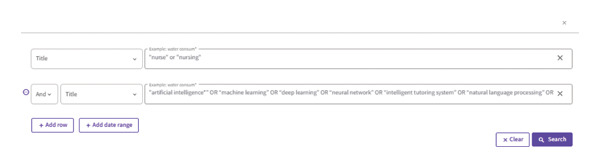
Search query string.

The WoS database was selected due to its reputation as one of the world’s most significant scientific citation indexing platforms [20]. No restrictions were applied regarding language or publication year. To identify relevant studies on the use of AI in nursing, a combination of advanced search techniques and keywords derived from the literature review was used. The search was conducted using the title field only to ensure high relevance and precision in capturing studies that explicitly focus on the intersection of AI and nursing. The keywords employed in the search process are presented in Figure 1. Similar to our findings, recent bibliometric analyses in healthcare‐related and interdisciplinary fields [19, 21] demonstrated the importance of mapping research hotspots and identified emerging technologies. These studies underscore the necessity of combining quantitative bibliometric indicators with network analysis to provide a comprehensive overview of scholarly landscapes.

The complete Boolean search query used in this study was as follows:

TI = (“nurse” OR “nursing”) AND TI = (“artificial intelligence” OR “machine learning” OR “deep learning” OR “neural network” OR “intelligent tutoring system” OR “natural language processing” OR “machine intelligence” OR “intelligent support” OR “chatbot” OR “automated tutor^∗^” OR “intelligent agent^∗^” OR “expert system^∗^” OR “intelligent system^∗^” OR “intelligent tutor^∗^” OR “robot” OR “sensing” OR “deep learning^∗^” OR “robotic^∗^”)

This query was specifically designed to retrieve studies where both nursing‐related terms and AI‐related terms appeared in the title, thereby maximizing the specificity and relevance of the dataset.

As shown in Figure 1, a total of 799 research articles were retrieved from the WoS database using the aforementioned search string. This query was designed to capture the most relevant studies addressing the intersection of AI and nursing, based on titles.

**FIGURE 2:**
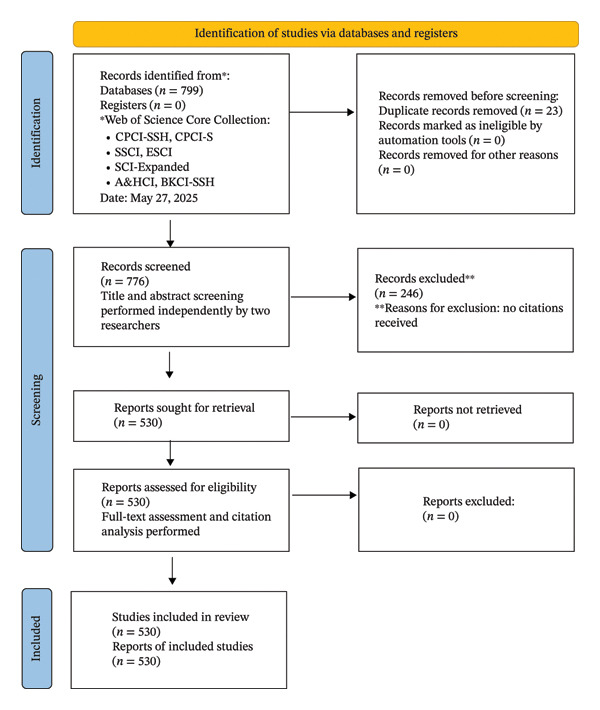
Flowchart for selection of studies.

A bibliometric methodology was adopted to analyze the scientific literature on AI in nursing, with study selection carried out through systematic identification, screening, eligibility evaluation, and inclusion procedures consistent with PRISMA 2020 standards. Figure 2 presents the PRISMA 2020 flow diagram [22], which outlines the systematic procedures followed in this study. In the identification phase, a comprehensive search of the WoS Core Collection (SSCI, SCI‐Expanded, AHCI, ESCI, CPCI‐S, CPCI‐SSH, and BKCI‐SSH) retrieved 799 records, from which 23 duplicates were removed through automated and manual procedures, leaving 776 unique publications. In the screening phase, two independent reviewers examined titles and abstracts, and 246 uncited studies were excluded based on the criterion requiring at least one citation to ensure interpretability in relationship‐based bibliometric mapping. Our exclusion strategy is based on the principles of relationship strength, which is the primary goal of bibliometric mapping, and on increasing map interpretability. Distance‐based mapping techniques such as VOSviewer aim to ensure that the distance between items reflects the strength of the relationship [12]. In this context, studies with zero citations (zero relationship strength) do not contribute significantly to the network structure. A total of 530 records met the basic relevance threshold. In the eligibility phase, all 530 records were accessible and evaluated in full text, and each was confirmed to align with the thematic and methodological scope of the review, resulting in no additional exclusions. In the inclusion phase, studies were retained if they were indexed in WoS; addressed AI within nursing practice, education, management, or research; and presented primary or secondary data relevant to nursing applications of AI. The process yielded a final dataset of 530 studies, forming the analytical foundation for mapping citation relationships, thematic clusters, and research trends in the field.

### 2.2. Data Cleaning and Standardization

Prior to analysis, extensive data cleaning and standardization procedures were conducted to ensure accuracy and consistency. Author name variations (e.g., “O’Connor S.” vs. “O′Connor, Siobhan” vs. “O’Connor, S.”) were manually standardized using a combination of automated matching in VOSviewer and manual verification through cross‐referencing with ORCID identifiers and institutional affiliations where available. Similarly, keyword normalization was performed to merge synonymous or closely related terms. For example, “AI,” “artificial intelligence,” and “machine intelligence” were consolidated under “artificial intelligence;” “robot,” “robotic,” and “robotics” were merged into “robotics.” This normalization process was conducted iteratively, with two researchers independently reviewing the keyword list and resolving discrepancies through discussion.

### 2.3. Ethical Considerations

Ethical review was not required for our study.

### 2.4. Validity, Reliability, and Rigor

All publication data were obtained from the WoS database in TXT format and exported into Excel. Subsequently, we employed the Excel built‐in text and statistical functions to ensure that only the data on the publications related to AI use in nursing were collected. Two researchers independently screened and selected the articles. Inter‐rater reliability was assessed using Cohen’s kappa coefficient (*κ* = 0.87), indicating a high level of agreement between reviewers [23]. Inclusion criteria required that the study (a) be indexed in the WoS Core Collection; (b) explicitly address AI in the context of nursing practice, management, education, or research; and (c) present primary or secondary data related to nursing applications of AI. Exclusion criteria included uncited publications and studies that did not focus on nursing‐related domains. These procedures ensured methodological transparency and minimized bias in study selection.

### 2.5. Data Analysis

Based on the defined conceptual parameters, the metadata of 799 publications were extracted through title‐based screening. Among them, 530 publications were included in the analysis as they had received at least one citation. The bibliographic data were downloaded in both tab‐delimited text and Excel formats. Initially, the extracted data were imported into VOSviewer, a widely‐used and freely available bibliometric analysis software. VOSviewer was utilized to analyze and visualize the relationships among authors, countries, journals, citations, and keywords [12].

Science mapping was conducted using VOSviewer (Version 1.6.20) and R Studio (Version 2025.09.1, Build 401). For keyword co‐occurrence analysis, the minimum number of keyword co‐occurrences was set to five (*n* = 5), ensuring that only relevant and frequently used terms were visualized. The normalization method applied was the LinLog/modularity technique, which allowed for more apparent separation between thematic clusters. In the coauthorship and country collaboration analyses, the fractional counting method was used to minimize bias arising from publications with multiple affiliations. Cluster resolution was set at 1.00, and a minimum threshold of two documents per author or country was adopted to ensure analytical robustness. The clustering algorithm employed modularity optimization to capture the structure of the bibliometric network accurately.

## 3. Findings

### 3.1. Descriptive Results

The distribution of the studies by publication type, language, and country is presented in Table 1. As shown in the table, the majority of publications on nursing and AI consist of peer‐reviewed journal articles (*N* = 346, 65.28%), followed by conference proceedings (*N* = 85, 16.04%) and review articles (*N* = 42, 7.92%). In terms of publication language, an overwhelming majority of the studies were published in English (*N* = 526, 99.25%), indicating the dominance of English as the primary language of scientific communication in this field. Regarding the country of origin, the highest number of publications originated from the United States (*N* = 147, 27.74%), followed by the People’s Republic of China (*N* = 108, 20.38%) and Japan (*N* = 51, 9.61%). These were followed by countries such as Germany, Saudi Arabia, and South Korea. These findings highlight that scientific production in the field of AI in nursing is predominantly global in scope and led primarily by technologically advanced countries.

**TABLE 1:** Distribution of studies by document type, language, and country.

		***N***	***f* (%)**

*Type of research*			
1	Article	346	65.28%
2	Proceeding paper	85	16.04%
3	Review article	42	7.92%
4	Editorial material	41	7.74%
5	Retracted publication	16	3.02%

*Language*			
1	English	526	99.25%
2	French	2	0.38%
3	Spanish	2	0.38%
4	Portuguese	2	0.38%

*Countries (Top 10)*			
1	USA	147	27.74%
2	People’s Republic of China	108	20.38%
3	Japan	51	9.62%
4	Germany	26	4.91%
5	Saudi Arabia	25	4.72%
6	South Korea	24	4.53%
7	England	19	3.58%
8	Taiwan	18	3.40%
9	Türkiye	18	3.40%
10	Others	94	17.74%

**FIGURE 3:**
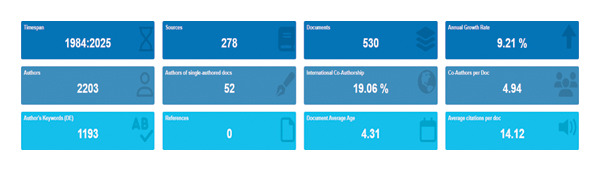
Bibliometric indicators of AI in nursing (1984–2025; through May 2025).

Figure 3 presents the main bibliometric indicators derived from 530 publications on AI in nursing indexed between 1984 and 2025 (through May 2025). These studies were published across 278 different sources, demonstrating an annual growth rate of 9.21%, which reflects the accelerating scholarly attention to AI applications in nursing and healthcare management. A total of 2203 authors contributed to this body of work, including 52 single‐authored papers, indicating a strong culture of collaboration in the field. The international coauthorship rate of 19.06% highlights a growing trend toward cross‐border research partnerships. On average, each publication involved 4.94 coauthors, suggesting multi‐institutional teamwork and increasing interdisciplinarity between nursing, computer science, and health informatics. The dataset contained 1193 unique author keywords, illustrating diverse research foci spanning from clinical care and robotics to decision‐support systems and nursing education. The average document age was 4.31 years, indicating that the majority of studies are recent and reflect contemporary developments in the discipline. Moreover, each publication received an average of 14.12 citations, demonstrating a solid and growing citation impact for this emerging field. Overall, these findings suggest that research on AI in nursing has expanded steadily over the past 4 decades, characterized by high collaboration intensity, strong international engagement, and increasing scholarly influence, underscoring its strategic relevance for innovation and evidence‐based management in modern nursing practice.

### 3.2. Publication Trends

**FIGURE 4:**
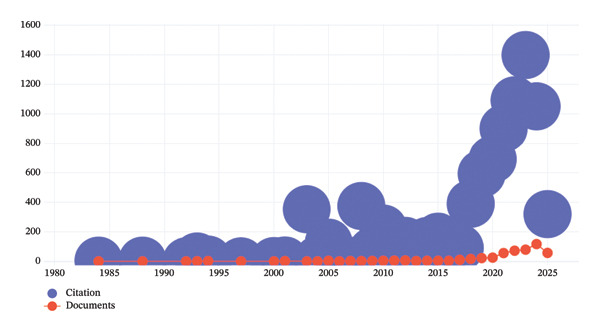
Change of article numbers and citations by year.

A total of 530 documents related to the intersection of nursing and AI, published between 1984 and 2025 (through May 2025), were analyzed within the scope of this research. The earliest publication in this field dates back to 1984, yet scientific output remained sporadic until the early 2000s. Between 1984 and 2007, the number of studies was limited, averaging fewer than five publications per year. However, a notable rise began in 2008, marking the early stage of increased scholarly attention to AI applications in healthcare. Between 2010 and 2018, the field gained steady momentum, with annual publication counts ranging between 5 and 23. This gradual upward trend was accompanied by a proportional increase in citations, indicating the growing academic and clinical relevance of the topic. Particularly, 2008 and 2003 stand out with unexpectedly high citation counts (376 and 354, respectively), likely due to seminal or highly referenced early studies that laid the groundwork for later research. A more rapid expansion occurred between 2019 and 2025, during which the volume of publications and citations reached unprecedented levels. The number of documents increased from 23 in 2019 to 118 in 2024, culminating in 58 publications within the first months of 2025. Similarly, citations rose dramatically, peaking at 1473 in 2023, reflecting a robust interest in integrating AI into nursing education, clinical decision‐making, and patient care. As seen in Figure 4, the steep increase in both publications and citations during the last 5 years demonstrates that AI has become a central research theme within nursing science. The decline in citations observed in 2024‐2025, despite high publication activity, may be attributed to the short time since publication, as recent articles have not yet accumulated sufficient citation counts. In fact, the metadata in the research include the first five months of 2025.

### 3.3. Bibliometric Findings

The number of publications, citation counts, and total link strength (TLS) values of authors included in the analyzed studies were examined. According to the VOSviewer manual, each coauthorship link is assigned a positive numerical strength; the higher the value, the stronger the collaborative relationship [24]. The TLS refers to the cumulative strength of an author’s coauthorship links with other researchers. Based on this, Table 2 presents the top 10 most cited authors who have contributed at least one publication in the indexed literature included in this study. As shown in Table 2, O’Connor S. is the most cited author, with 455 citations from a total of 8 publications. She is followed by Topaz, M. (9 publications, 350 citations) and Pruinelli, L. (5 publications, 260 citations). Notably, the author with the highest TLS is Guo S., who, despite having 218 citations from 9 publications, stands out with a TLS of 32, indicating extensive collaborative engagement across a wide network of researchers. Similarly, Hirano S. and Nakashima H. also appear as central figures in multiauthor collaborative structures based on their high TLS values. The other authors listed also contribute significantly to the field in terms of both productivity and scholarly impact.

**TABLE 2:** The top 10 prolific authors based on the total number of publications.

Rank	Author	Documents	Citations	TLS
1	O’Connor S.	8	455	19
2	Topaz M.	9	350	18
3	Pruinelli L.	5	260	15
4	Mukai T.	10	247	30
5	Bakken S.	6	227	16
6	Guo S.	9	218	32
7	Hirano S.	5	215	24
8	Nakashima H.	5	203	21
9	Liu Y.	5	96	13
10	Li Y.T.	5	88	2

Table 3 presents the institutions affiliated with the most highly cited authors, ranked by total citation count, number of publications, and TLS. As shown in Table 3, Columbia University stands out as the leading institution with 14 publications and 474 citations. It is followed by the University of Manchester (5 publications and 410 citations) and the University of Minnesota (7 publications and 321 citations). Researchers affiliated with Columbia University not only demonstrate high productivity but also hold a central position in terms of collaborative strength (TLS = 26). Similarly, the University of Minnesota exhibits both a high level of citation impact and a substantial TLS value (23), indicating its strong involvement in international research networks. Institutions such as the National University of Singapore, Taipei Medical University, and the National Taiwan University of Science and Technology also contribute significantly to the literature, although with comparatively lower collaborative intensity. Moreover, universities such as the University of Tokyo, Purdue University, and the University of Pennsylvania appear in the top ranks with a relatively smaller number of publications. In terms of TLS, Columbia University is followed by institutions such as the University of Minnesota and the University of Pennsylvania, which also engage in substantial coauthorship activities. In contrast, institutions such as Ewha Womans University, despite receiving a noteworthy number of citations, display limited collaborative connections, suggesting lower engagement in coauthored research. These findings underscore the dual importance of scholarly output and research collaboration in assessing the academic visibility and structural influence of institutions in the domain of AI in nursing.

**TABLE 3:** The top 10 institutions based on the total number of publications.

Rank	Institution	Country affiliation	Documents	Citations	TLS
1	Columbia University	USA	14	474	26
2	University of Manchester	UK	5	410	13
3	University of Minnesota	USA	7	321	23
4	National University of Singapore	Singapore	6	215	7
5	Taipei Medical University	Taiwan	7	185	6
6	National Taiwan University of Science and Technology	Taiwan	5	142	7
7	University of Pennsylvania	USA	5	97	8
8	Purdue University	USA	5	88	0
9	University of Tokyo	Japan	9	56	1
10	Ewha Womans University	South Korea	5	49	0

As presented in Table 4, the distribution of publications across journals shows that Nurse Education in Practice emerges as the most influential outlet in the intersection of nursing and AI. With 16 publications and 452 citations, it ranks first in terms of scholarly influence. It also shows a strong collaborative profile with a TLS of 13. The journal frequently features studies focused on innovative approaches to nursing education and the integration of technology into clinical training. As of 2024, its impact factor (IF) is 3.3, reflecting a moderate‐to‐high level of academic influence. Following closely is Nurse Education Today, which has published 13 articles and received 261 citations, although it exhibits a more limited TLS of 6. This journal emphasizes pedagogical strategies and educational methodologies in nursing curricula. The Journal of Clinical Nursing ranks third (8 publications, 241 citations, and TLS = 16), publishing interdisciplinary work that bridges clinical practice and academic nursing scholarship. Overall, the top‐ranked journals by citation count predominantly focus on nursing and health informatics. For instance, the Journal of Medical Internet Research (6 publications, 231 citations, and TLS = 5) and Computers Informatics Nursing (CIN) (19 publications, 192 citations, and TLS = 12) publish widely on digital health, e‐health applications, and clinical decision‐support technologies. In contrast, journals such as the Journal of Healthcare Engineering and the Cureus Journal of Medical Science show relatively lower citation and collaboration metrics. When examining IFs, the Journal of Medical Internet Research holds the highest value (IF = 5.0), followed by the Journal of Nursing Management (IF = 3.7) and the Nurse Education Today (IF = 3.6). At the bottom of the list, the Cureus Journal of Medical Science records 60 citations, a TLS of 1, and an IF of 1.1, reflecting its more limited visibility and collaborative engagement in the field.

**TABLE 4:** The top 10 journals with the most publications and citations.

Rank	Journal title	Documents	Citations	TLS	IF^∗^
1	Nurse Education in Practice	16	452	13	3.3
2	Nurse Education Today	13	261	6	3.6
3	Journal of Clinical Nursing	8	241	16	3.2
4	Journal of Medical Internet Research	6	224	1	5.8
5	Computers Informatics Nursing (CIN)	19	192	12	1.3
6	Journal of Nursing Management	14	191	19	3.7
7	Healthcare	8	108	3	2.4
8	BMC Nursing	12	105	3	3.1
9	Journal of Healthcare Engineering	13	79	1	1.7
10	Cureus Journal of Medical Science	5	60	1	1.1

^∗^Impact factor (IF): 2024 values retrieved from the official websites of the respective journals.

As shown in Table 5, the United States is the leading contributor to AI–nursing research, producing 147 publications and 2855 citations and exhibiting the highest TLS (TLS = 336). This pattern reflects not only the country’s high scientific productivity but also its strong integration into international research networks. The United Kingdom follows as the second‐most influential country, with 19 publications, 788 citations, and a substantial TLS of 167, indicating dense and sustained coauthorship ties with global partners. The prominence of the United Kingdom is also evident at the institutional level. As noted in earlier tables, approximately one‐third of the top‐ranked universities are located in the United Kingdom, underscoring the alignment between national and institutional contributions. The People’s Republic of China ranks third with 108 publications, 705 citations, and a TLS of 133, demonstrating a rapidly growing and internationally connected research community. Other notable contributors include Japan (51 publications, 559 citations, and TLS = 67), Canada (14 publications, 418 citations, and TLS = 110), and Germany (26 publications, 386 citations, and TLS = 75). In particular, Canada’s relatively modest output paired with a high TLS indicates a strong emphasis on international collaboration. Countries such as Finland, Denmark, Taiwan, and Australia appear in the lower half of the ranking. Although their publication counts are smaller, their citation impact and collaboration metrics signal meaningful engagement in the field. Australia, for example, ranks tenth with 16 publications, 237 citations, and a TLS of 56, reflecting its significant international connectivity relative to output volume. A notable finding is the absence of Turkey from the list of the ten most cited countries, despite producing a considerable number of publications on AI in nursing (see Table 1). This discrepancy may reflect challenges related to global visibility, citation performance, or the intensity of international collaboration. Enhancing cross‐national partnerships and citation impact may therefore represent strategic priorities for researchers in Turkey.

**TABLE 5:** The top 10 most prolific countries/regions based on the total number of publications.

Rank	Country	Documents	Citations	TLS
1	USA	147	2855	336
2	England	19	788	167
3	People’s Republic of China	108	705	133
4	Japan	51	559	67
5	Canada	14	418	110
6	Germany	26	386	75
7	Finland	8	325	81
8	Denmark	5	316	64
9	Taiwan	18	287	106
10	Australia	16	237	56

As shown in Table 6, the most highly cited studies at the intersection of nursing and AI primarily address robotic care assistants, AI‐supported educational platforms, and communication technologies. The most cited publication is Pineau et al. [25], dealing with the challenges of implementing robotic assistants in nursing homes, which currently boasts 347 citations. If the citation impact over time is taken into account, the average citation annual values clearly point to the emergence of O’Connor [26] with the highest average annual citation impact of 75.25, marking the increased scholarly concern over open platforms of AI, particularly from the perspective of nursing education. There appears to be a definite rise in the citations over the past few years. Publications from the year 2021, such as Ronquillo et al. [27], with 49 citations, and von Gerich et al. [28], with 56 citations, have promptly attained significant status, indicating a shift in the area of focus from several fundamental aspects of technology implementation to more contemporary aspects of AI in nursing education. Innovations such as mobile chatbot technology by Chang et al. [29] and the virtual counseling application by Shorey et al. [30] have gradually received increased scholarly focus, primarily because of the positive outcomes observed in terms of learning outcomes among nursing students. The increased citation rates over the past few years, with respect to recent publications, depict a rapidly evolving literature on the topic, where recent publications easily register significant scholarly impact within a short period of time.

**TABLE 6:** Descriptive characteristics of the top 10 most cited nursing and AI studies.

Rank	Title	Authors/year published	Total citations	Average per year	Number of citations in the last 5 years				
					2021	2022	2023	2024	2025
1	Towards robotic assistants in nursing homes: Challenges and results	Pineau et al. [25]	347	15.09	21	20	10	6	4
2	Open artificial intelligence platforms in nursing education: Tools for academic progress or abuse?	O′Connor [26]	300	75.25	0	0	167	114	20
3	Development of a Nursing‐Care Assistant Robot RIBA That Can Lift a Human in Its Arms	Mukai et al. [31]	161	10.06	14	16	13	13	5
4	Artificial intelligence in nursing: Priorities and opportunities from an international invitational think‐tank of the Nursing and Artificial Intelligence Leadership Collaborative	Ronquillo et al. [27]	124	24.8	1	24	18	49	31
5	Artificial Intelligence–based technologies in nursing: A scoping literature review of the evidence	von Gerich et al. [28]	113	28.25	0	17	20	56	20
6	Promoting students’ learning achievement and self‐efficacy: A mobile chatbot approach for nursing training	Chang et al. [29]	105	21.2	1	9	27	44	25
7	A Virtual Counseling Application Using Artificial Intelligence for Communication Skills Training in Nursing Education: Development Study	Shorey et al. [30]	96	13.71	9	20	24	22	13
8	Application Scenarios for Artificial Intelligence in Nursing Care: Rapid Review	Seibert et al. [32]	94	18.8	0	12	21	39	22
9	Futurism in nursing: Technology, robotics, and the fundamentals of care	Archibald et al. [33]	93	11.63	14	19	21	10	6
10	Robotics in Nursing: A Scoping Review	Maalouf et al. [6]	80	10	15	19	18	17	6

### 3.4. Coauthorship: Authors

**FIGURE 5:**
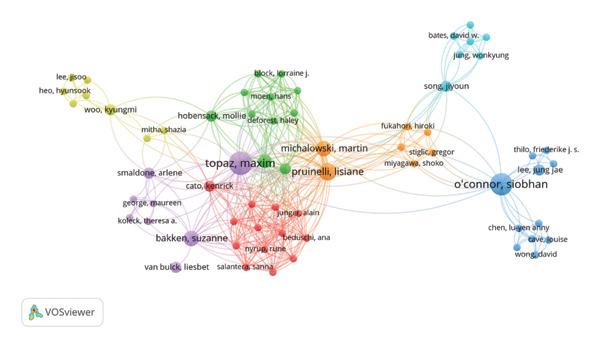
Coauthorship network map.

Figure 5 illustrates a coauthorship network visualized using VOSviewer, representing collaborative relationships among authors in the selected research domain. Each node corresponds to an individual author, while the edges signify coauthored publications. Node size reflects the author’s publication count and citation impact, whereas node color indicates different clusters that represent distinct collaboration communities (e.g., blue = O’Connor group, red = Peltonen group, green = Chu and Prunelli group, and yellow = Topaz cluster). Color‐coding helps distinguish research partnerships and institutional associations. Specifically, there is Siobhan O’Connor who has a remarkable record of 8 publications and 455 citations and a TLS of 18. Only Maxim Topaz has a TLS of 54 and has been involved in a total of 9 publications and has a total of 350 citations. The TLS gives a measure of the strength of coauthorship associations of each contributing author. The TLS is assessed based on the number of connections and indirectly measures how connected each contributing author is. The contributing authors in this case include Lisiane Pruinelli who has a total of 260 citations and a TLS of 42 after contributing to a total of 5 publications. Then, there is Laura‐Maria Peltonen who has a total of 239 citations and a TLS of 32 after contributing to a total of 3 publications. The other contributor is Charlene H. Chu, who has a total of 237 citations and a TLS of 31 after contributing to a total of 2 publications. The last one is Suzanne Bakken who has a total of 189 citations and a TLS of 25 after contributing to a total of 4 publications. The different link colors give different identities in terms of themes. The blue link group is identified by O’Connor and has a focus on AI and nursing educations. The orange link represents clinical machine learning applications. The yellow link is identified by simulation technology and chatbot‐based nursing educations. The green link has a focus on clinical workflow analysis and electronic healthcare‐based operational analytics. The last one is the purple link identified by nursing informatics. The link has a special focus on NLP. The red link is one of the most densely connected communities and has a focus on robotics nursing.

### 3.5. Co‐Occurrence: Keywords

**FIGURE 6:**
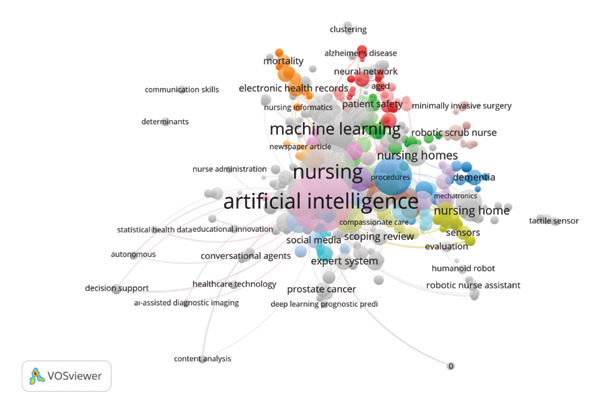
Network map of the most frequently used keywords.

Figure 6 presents a co‐occurrence analysis of author keywords used in publications within the selected field. Each node represents a distinct keyword, with node size reflecting the number of occurrences, and links indicating the frequency of co‐occurrence with other keywords. The colors show clusters of related keywords organized around themes, with different colors representing different clusters of themes identified by unique colors, where keywords having the same color represent their strong thematic associations and frequent co‐occurrences in published articles. The close location of keywords represents their strong conceptual associations, where keywords placed close to each other have frequent co‐occurrences. Colors represent clusters of related keywords organized around themes categorized by different colors. The close location of keywords represents their strength of conceptual associations. Among the keywords having the highest link strength in the network map, “artificial intelligence” (keyword with 113 occurrences with TLS of 484) dominates this area with a focus related to “nursing” (keyword with 70 with TLS of 368) and “machine learning” (keyword with 40 with TLS of 184). On frequent co‐occurrences of different keywords with link strength, “nursing education” (keyword with 30 with 126 link strength) has the topmost priority with respect to “robotics” (keyword with 26 with 138 link strength) and “deep learning” (keyword with 18 with 89 link strength). The TLS has been found to be highest with respect to this network map’s “artificial intelligence” (keyword with 113 with TLS of 484) with respect to themes related to nursing, machine learning, robotics, natural processing, and health care. In this respect, TLS gives a representation of strong conceptual link co‐occurrences of this specific uniquely identified keyword with respect to other keywords within this network map. The map carries fruitful representation of integration of different AI technologies with nursing practices and education.

### 3.6. Citations by Country (TLS)

**FIGURE 7:**
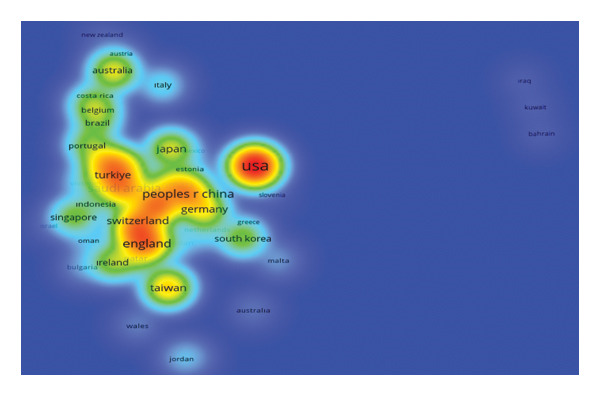
Density map of the most productive countries.

As illustrated in Figure 7, the global distribution of citations and collaborative link strengths reveals clear geographic patterns in the development of AI–nursing research. This is shown through the use of the color standard (red corresponds to the highest density, yellow or green corresponds to moderate activity, and blue corresponds to the lowest density). According to the results, the United States is the leading region in the study of AI and nursing. It has the highest number of publications (147), citations (2855), and the best TLS (385). These indicators depict the significant role the region plays in the body of knowledge. England is the second leading region; it has 19 publications, 788 citations, and TLS = 179. Third comes the People’s Republic of China; it produced 108 publications, 705 citations, and TLS = 144. Other leading nations that produced significant AI and nursing research output and impacts include Japan, Canada, and Germany. Collaboration intensity patterns bring in more complexities. A set of countries displays strong international collaboration despite the moderate rate of published papers. For example, Saudi Arabia with 25 papers showed a TLS of 141, symbolizing strong collaborative connections. Similarly, Switzerland with a minimum of five papers showed a TLS of 103, symbolizing strong integration into the global coauthorship network. Turkey, with 18 papers and 62 citations, showed a TLS of 88, symbolizing its increasing participation in international scientific collaborations. These results, together, symbolize that the domain of AI and nursing is situated in a very different global ecosystem, which is both diversified and increasingly interconnected. Though some countries lead in the overall volume as well as citation impact, others have critical roles in serving as facilitators for cooperation among different international partners in the domain’s overall international know‐how exchange.

### 3.7. Citation: Authors

**FIGURE 8:**
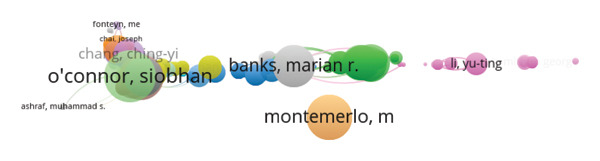
Network map of the most cited authors.

In Figure 8, the coauthorship network among the researchers emerges through the use of the total number of citations and the TLS among the publications related to AI and nursing. Every node in the figure represents the individual researchers. Additionally, the size of the nodes is measured by the citations, while the thickness corresponds to the bonds among the coauthors. Colors among the groups illustrate the coauthorship among the researchers. From the analysis, the author who appears to be the central author in the coauthorship network is Maxim Topaz, who has authored 9 publications, received 350 citations, and has the highest TLS of 321. In addition, the other authors who show significant academic influence, apart from the previous ones, are Lisiane Pruinelli, who has 5 publications, 260 citations, and a TLS of 238, and Siobhan O’Connor, who has 8 publications, 455 citations, and a TLS of 228. However, the author who has the overall highest citation number is Siobhan O’Connor. Other important individuals who score highly in the value of their TLS measures include Laura‐Maria Peltonen, who has 3 publications and 239 citations and a TLS of 222; Martin Michalowski, who has 4 publications and 259 citations and a TLS of 221; and Suzanne Bakken, who has 4 publications and 189 citations and a TLS of 220. On the other hand, some authors can be defined by the large amount of influence exerted by one of their publications. Montemerlo M., Pollack M., Roy N., Thrun S., and Pineau J. published only one paper and received 347 citations. This occurred despite having low collaboration ties, as evidenced by their low TLS values of 14. Others include Marian R. Banks, William A. Banks, and Lisa M. Willoughby, who each received 308 citations for one of their publications and had low collaboration ties as measured by their high TLS values of 15. In sum, the networks demonstrate the presence of both highly central and cooperative researchers as well as heavily cited peripheral researchers. TLS is found to be a complex metric that not only indicates the coauthorship graph density but also the author’s structure in the research environment. These findings highlight the fact that the scientific production in the field of “Artificial Intelligence in Nursing” is a highly cooperative activity that has high‐impact individual production as well as comprehensive research networks.

### 3.8. Citation: Countries

**FIGURE 9:**
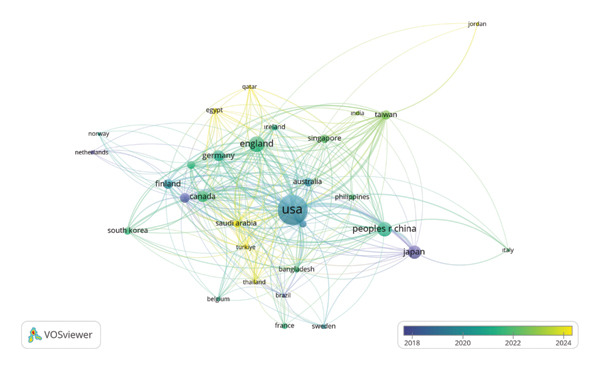
Network map of the most cited countries over the years.

Figure 9 shows the pattern of the international cooperation map in nursing and AI studies, which feature a highly dense and centralized pattern. The main actor in the international cooperation map is the United States, represented as the foremost producer of knowledge in the field and the most centralized hub in the global cooperation map, with 139 papers, 2850 citations, and a TLS of 6804. The second main actor in the international cooperation map is the People’s Republic of China, which is the foremost leader in the Asian grouping and the second‐most productive actor in the map with 102 papers and a citation strength of 3309. The other actors in the international map are the United Kingdom, Canada, Finland, Germany, Japan, and South Korea. The color spectrum reflecting the time span of 2018–2024 depicts the dynamic process of publication output over time. The historically productive nations of the United Kingdom, Finland, Canada, and Germany are colored bluish; in contrast, the countries of Turkey, Qatar, Egypt, Jordan, Taiwan, and Saudi Arabia are colored green–yellow, pointing to growing activity over the more recent years. The pattern indicates a geographical spread of research on AI in nursing and a quicker research pace in the regions of Asia and the Near East after the year 2021. Recently, the Saudi Arabia (25 publications, 174 citations, and TLS 4216) and Turkey (18 publications, 62 citations, and TLS 2683) regimes particularly exhibit high TLS values. Despite their lower publication and citation values, the two regimes exhibit high engagement in the global collaborative efforts through their robust engagement in the form of networks. Additionally, the regime of Finland just by publishing eight studies achieves a qualitatively significant status because of its high number of citations (325). In conclusion, the structure of the network indicates that the research studies related to nursing and AI entail high collaborative engagement globally as well as within the regional and globally integrated regimes.

### 3.9. Thematic Map

**FIGURE 10:**
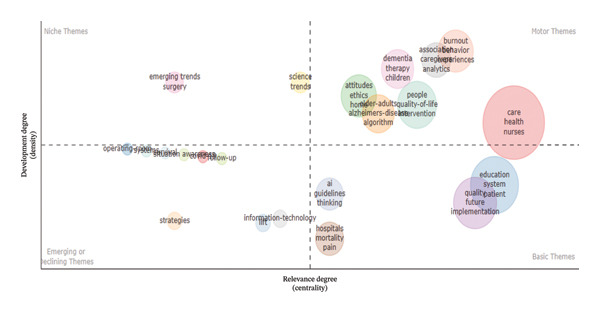
Thematic map analysis.

Figure 10 shows the thematic map portraying the strategic positioning of research on AI in nursing. This thematic map identifies the quadrants on the axes given by the concepts of development (density) and relevance (centrality). Thematic concepts identified in the upper‐right quadrant include Motor Themes: care, health, and nurses. These concepts represent the well‐developed and highly central ideas in the context of AI in nursing. They show the positioning of AI in nursing practices and how the envelope could be expanded in the context of patient care management and risk analysis. These concepts reveal the managerial role associated with the application of AI in the optimization and supervision of nursing staff. Thematic concepts in the lower‐right quadrant include Basic Themes: education, system, quality, patient, and implementation. These domains represent fundamental yet evolving areas that form the conceptual foundation for AI adoption in nursing. Their positioning reflects increasing managerial focus on integrating AI into quality improvement systems, patient safety initiatives, and the digital transformation of nursing education and training. The upper‐left quadrant includes Niche Themes such as “emerging trends,” “surgery,” and “science trends,” which denote specialized or exploratory subfields characterized by high internal cohesion but limited overall impact. These areas frequently serve as innovation hubs that may influence advanced practice nursing and clinical leadership in the future. In contrast, the lower‐left quadrant contains Emerging or Declining Themes such as “information technology,” “strategies,” and “life.” This positioning indicates transitional topics that are either evolving into more influential areas or diminishing as research priorities change. The coword network table further supports this thematic structure, showing that “care” (occurrences = 37), “health” (27), and “nurses” (16) possess the highest betweenness‐centrality values, which confirms their integrative function in linking subthemes such as “risk,” “prediction,” “technology,” and “management.” Overall, these findings indicate that research on AI in nursing is increasingly focused on clinical excellence, data‐driven managerial decision‐making, and innovation in patient‐centered care, while educational and implementation domains continue to offer significant opportunities for development and leadership.

## 4. Discussion

This 40‐year bibliometric analysis provides a comprehensive overview of the evolution and current landscape of scholarly publications on AI in nursing. Although early studies appeared in the 1980s [34, 35], the field remained relatively quiet for several decades. A marked transformation occurred after 2018, when both publication and citation counts increased rapidly. Similar turning points have been reported in broader areas of health‐related AI [14] and in digital health fields such as telemedicine [36]. This parallel timing suggests that nursing research did not evolve in isolation. Instead, it responded to global developments such as the expansion of data infrastructures, the widespread availability of machine learning tools, and the growing interest in interdisciplinary collaboration.

The geographic distribution of publications shows a highly concentrated research environment. The United States produced nearly half of the studies in the dataset, while contributions from low‐ and middle‐income countries remained limited. This uneven pattern resembles earlier findings in global health research, where digital capacity and access to large datasets strongly influence output [37]. The same structural imbalance appears in patterns of coauthorship. Only a small proportion of the publications involved cross‐country collaboration, and the main authors operated within relatively separated networks. Even influential researchers such as O’Connor (TLS 19) and Topaz (TLS 54) worked within clusters that overlapped only minimally. These patterns demonstrate that AI in nursing has not yet developed the type of international research networks that help to establish shared standards and stable thematic areas.

Citation patterns also reveal an important shift. The most frequently cited work in the dataset, Pineau et al. [25], focused on robotic assistants, which reflects an earlier period when automation and expert systems dominated discussions. However, these themes declined in visibility during the last 5 years. At the same time, studies on AI in nursing education gained considerable momentum, particularly the study by O’Connor [26], which recorded the highest annual citation average. Publications on adaptive learning systems and digital instruction tools show a similar rise [29, 38]. This growing scholarly interest aligns with broad, multidisciplinary findings indicating that nursing, dental, and medical students generally hold positive attitudes toward AI use (pooled attitude proportion of 65%) [39]. However, this optimism coexists with a significant knowledge gap, as only a moderate proportion of students (44%) exhibit adequate theoretical or practical knowledge of AI principles [39]. This transition resembles developments observed in digital health, where interest gradually moved from mechanical systems to cognitive and data‐driven applications [14, 36, 40].

Although interest in these emerging areas is growing, the overall thematic structure remains dispersed. No single subdomain, aside from the general category of AI, has established dominant influence. This fragmentation is a characteristic feature of research areas that are still forming their conceptual boundaries. Comparable developmental patterns have been documented in fields such as telemedicine and digital public health during their early expansion periods [36, 40]. The same tendency is visible in how managerial and ethical topics appear in the network. These themes do not yet form strong clusters, even though recent publications increasingly emphasize the need to align AI technologies with leadership practices, institutional expectations and workforce readiness [41–43]. This need for ethical grounding is echoed across specialized medical domains where AI application raises concerns regarding algorithmic bias, the “black‐box” nature of models, patient privacy, and the important requirement for explainable AI to ensure clinical acceptance and patient safety [15, 18]. Studies on nursing management show that managers mostly view AI as supportive, while concerns related to ethics and safety remain influential [44, 45]. These findings help explain why managerial and ethical clusters appear relatively late and in limited form within the bibliometric map.

Taken together, the results indicate that AI in nursing is moving through a phase of rapid but uneven development. The sharp increase in publications since 2018, the limited global integration of research teams, the thematic fragmentation, and the lack of widely cited conceptual frameworks all point to a field that is expanding quickly but have not yet formed a unified research identity. Prior bibliometric studies focused on narrower scopes or shorter‐time periods [5, 46], whereas this long‐term perspective clarifies both the strengths and the gaps that shape the field. The synthesis presented here shows that AI research in nursing is progressing, but it requires stronger conceptual foundations, broader international collaboration, and more sustained attention to ethical and managerial dimensions if it is to develop into a coherent and mature domain.

## 5. Conclusion

This 40‐year bibliometric review of global scholarly output at the intersection of AI and nursing reveals the evolution and contemporary trends of this growing field. While AI is not new to healthcare, its application in nursing research has seen remarkable growth in recent years. Most recent contributions originate from developed countries and focus heavily on nursing informatics. Our analysis made visible the dominant research themes (e.g., patient safety, mental health, and next‐generation chatbots) and collaborative networks. In doing so, this study contributes to a unique and comprehensive perspective to the literature and identifies gaps and opportunities for future inquiry. As the study to map AI in nursing over such an extended period using WoS data, our findings offer strategic insights for future research, education, and policymaking. Moreover, the findings highlight the pressing need for standardized taxonomies and ontologies within AI–nursing research. Establishing a common framework for concepts, terminologies, and data categorization will enhance comparability across studies, facilitate interoperability between datasets, and support cumulative knowledge‐building in the field [19]. To strengthen future research, the development of a publicly accessible, open‐source AI‐nursing dataset or registry is strongly recommended. Such a repository would enable continuous bibliometric updates, global collaboration, and transparency in AI‐driven nursing innovation. Similar to our findings, recent bibliometric analyses in healthcare‐related and interdisciplinary fields report comparable trends [47].

## 6. Future Research Directions and Implications

The findings of this 40‐year bibliometric review point to several priorities for strengthening research and practice at the intersection of AI and nursing. Although publication activity has increased rapidly, robust evidence regarding the impact of AI on patient outcomes, workflow efficiency, and clinical decision‐making is still limited. Future research should therefore move toward mixed method and longitudinal designs that evaluate AI tools in routine clinical settings. Such studies would support the validation of decision support systems, clarify their contribution to patient safety, and reveal how they can be integrated into day‐to‐day nursing work.

The recent literature in nursing management emphasizes that successful adoption of AI depends on the alignment of technological capacity, workforce competencies, and ethical leadership [42, 43]. Strengthening AI literacy, encouraging innovation‐oriented organizational cultures, and promoting collaboration between clinicians and data scientists are essential steps for bridging this gap. These efforts can help ensure that technological progress translates into measurable gains in safety, efficiency, and care quality.

The bibliometric maps also highlight significant geographic disparities, with limited contributions from low‐ and middle‐income countries. Building a more equitable global research landscape will require targeted investment in digital infrastructure and the formation of international partnerships capable of evaluating AI applications across different health‐system contexts. The thematic clusters identified in this study may guide researchers in selecting appropriate journals, funding sources, and collaborative networks.

In clinical practice, AI is increasingly used for patient monitoring [48], risk prediction [49], documentation analysis [50], and conversational support [29, 30]. These areas correspond closely with the clusters identified in the keyword co‐occurrence network. To use such tools responsibly, nurses must be prepared to interpret AI outputs, recognize potential errors, and integrate algorithmic insights into their clinical reasoning without compromising professional judgment. Nursing curricula and continuing education programs should therefore include foundational knowledge in AI, data literacy, and ethical and legal issues such as transparency, privacy, and algorithmic bias.

## 7. Limitations

While this bibliometric analysis offers valuable insights, several limitations should be acknowledged. First, only articles indexed in the WOS were analyzed; publications in nonindexed or regional journals were excluded. Second, the exclusion of uncited papers, although methodologically justified to maintain citation consistency, may have underrepresented emerging research trends. Third, bibliometric methods cannot fully capture the contextual, ethical, or organizational nuances of AI adoption identified in qualitative nursing‐management studies. Fourth, the exclusion strategy carries the potential to bias the dataset toward more relationally mature studies. Therefore, the generated bibliometric map cannot claim to be a comprehensive representation of all publications in the relevant field. Its ability to accurately represent the most current or emerging themes of research is limited, particularly because publications that have not yet begun to gain citations or are relatively new to the field are excluded. Finally, despite careful data cleaning and normalization, variations in author names and institutional affiliations may have introduced minor inaccuracies in network analysis. These limitations should be considered when interpreting results and may guide the design of future, mixed‐method follow‐up studies integrating bibliometrics with qualitative inquiry.

## Author Contributions

Conceptualization, methodology, data curation, and formal analysis: M.D. and F.E.; investigation and visualization: F.E.; supervision and validation: M.D.; writing–original draft preparation: F.E.; writing–review and editing: M.D. and F.E.

## Funding

This research was supported by the Scientific Research Projects Coordination Unit of Fırat University (Grant No. EF.25.15).

## Conflicts of Interest

The authors declare no conflicts of interest.

## Data Availability

The data that support the findings of this study are available from the corresponding author upon reasonable request.
